# Micronutrients in polycystic ovary syndrome: molecular pathways, deficiencies, and therapeutic potential

**DOI:** 10.3389/fendo.2026.1766838

**Published:** 2026-02-10

**Authors:** Madhumitha Natarajan, Shweatha H. E., Raghu Nataraj

**Affiliations:** 1 Division of Molecular Biology, School of Life Sciences, Jagadguru Shri Shivaratreeshwara (JSS) Academy of Higher Education and Research, Mysore, Karnataka, India; 2 Department of Nutrition and Dietetics, Jagadguru Shri Shivaratreeshwara (JSS) Academy of Higher Education and Research, Mysore, Karnataka, India; 3 Division of Molecular Biology, Faculty of Life Sciences, Jagadguru Shri Shivaratreeshwara (JSS) Academy of Higher Education and Research, Mysore, Karnataka, India

**Keywords:** inflammation, insulin resistance, micronutrients, oxidative stress, polycystic ovary syndrome, precision nutrition

## Abstract

Polycystic Ovary Syndrome (PCOS) represents a complex endocrine–metabolic condition which presents with hyperandrogenism and anovulation together with insulin resistance and chronic low-grade inflammation affecting 21% of women during their reproductive years globally. Nutrition has always played a pivotal role in managing PCOS. Emerging evidence demonstrates that micronutrients play an essential part in regulating molecular processes that drive the pathophysiology of PCOS. The deficiency of micronutrients exacerbates insulin resistance, oxidative stress and hormonal dysregulation through their negative impact on PI3K/Akt, NF-κB and Nrf2 and steroidogenic enzyme signaling pathways, all of which play a key role in the pathophysiology of PCOS. This review synthesizes a comprehensive analysis of scientific findings which demonstrate how micronutrient levels influence the regulation of insulin function, inflammatory reactions, oxidative balance, methylation activities and ovarian health in PCOS patients. It also evaluates the potential advantages of targeted micronutrient supplementation used alongside standard management strategies, considering factors such as bioavailability and nutrigenomics, while emphasizing the need for robust large-scale randomized clinical trials. Overall, a molecularly targeted approach to micronutrients represents an emerging precision-nutrition strategy aimed at improving metabolic, reproductive, and inflammatory outcomes in women with PCOS.

## Introduction

**Figure 1:**
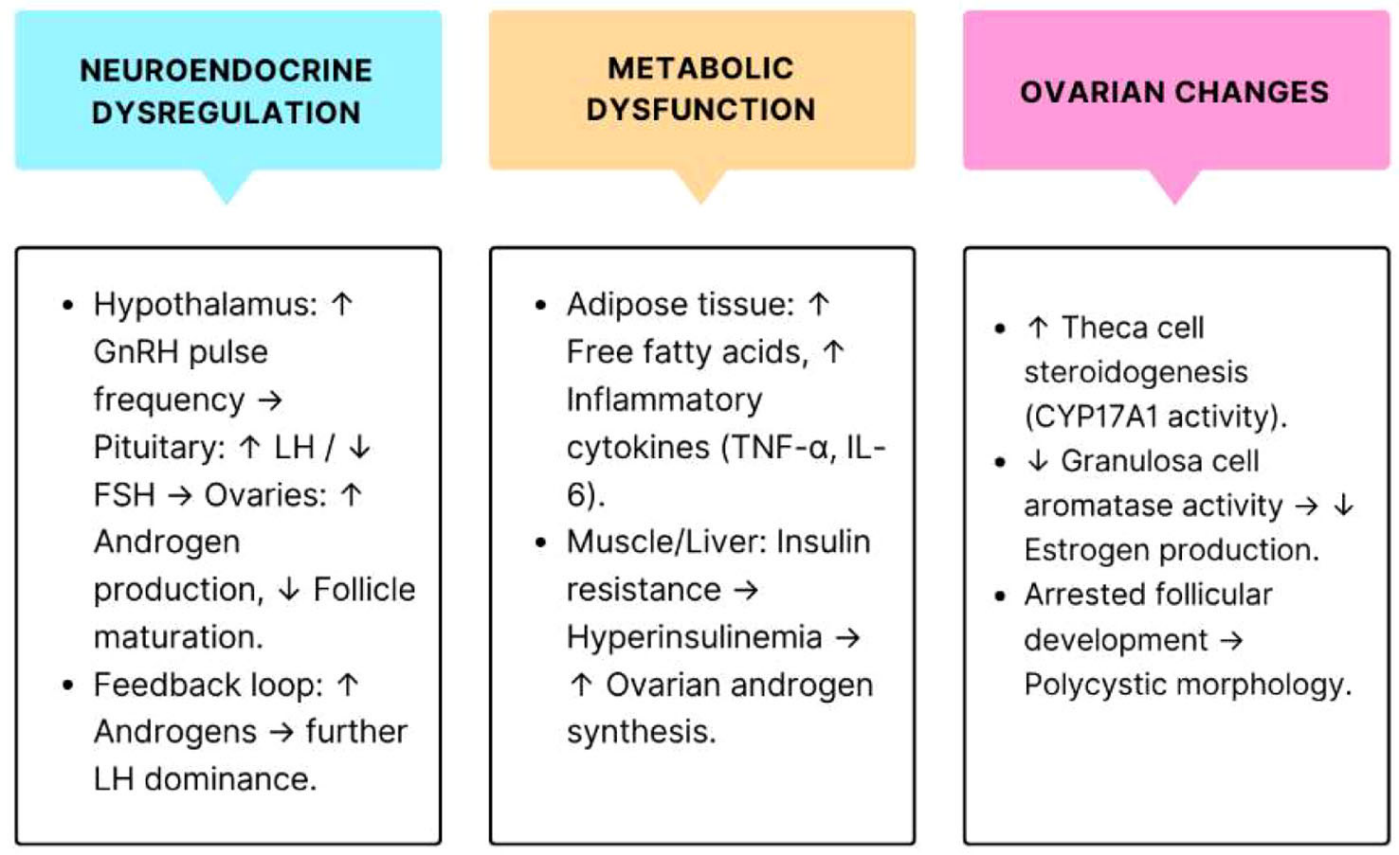
Pathophysiology of polycystic ovary syndrome: neuroendocrine, metabolic, and ovarian dysregulation. GnRH, Gonadotropin-Releasing Hormone; LH, Luteinizing Hormone; FSH, Follicle-Stimulating Hormone; TNF-α, Tumor Necrosis Factor Alpha; IL-6, Interleukin 6. CYP17A1, Cytochrome P450 Family 17 Subfamily A Member 1; Symbols: ↑, Increased; High, ↓, Decreased/Low.

Polycystic Ovary Syndrome (PCOS) stands as one of the most prevalent endocrine–metabolic conditions affecting women during their reproductive years at a rate of 21% worldwide and varies from 7% to 20% in Indian women based on different diagnostic standards. Defined by hyperandrogenism, ovulatory dysfunction, insulin resistance, and chronic low-grade inflammation, PCOS, a common endocrine disorder in reproductive aged women causes irregular periods, infertility, excess androgens which collectively contributes toward long-term risks for serious conditions like type 2 diabetes, cardiovascular diseases, sleep apnea, depression, endometrial cancer and other reproductive complications underscoring the need for a comprehensive, multifaceted management strategy (1, 2) (**Figure 1**).

The development and management of PCOS are strongly influenced by overall nutritional status, including dietary intake patterns and micronutrient composition. The composition of an individual’s diet affects important molecular pathways that regulate insulin signaling together with oxidative stress, inflammation and steroidogenesis. A diet composed of whole minimally processed foods with proper macronutrient proportions and sufficient micronutrient intake helps enhance insulin sensitivity along with hormonal balance and ovulatory function (3–5).

In accordance with epidemiological data, PCOS is common throughout the world, but its metabolic severity and phenotypic expression differ in developed and developing countries (6). While women in developing countries may display lean PCOS phenotypes with severe reproductive dysfunction, women in industrialized countries frequently present with obesity-associated insulin resistance and metabolic syndrome (7). High glycaemic load, ultra-processed foods, micronutrient-poor diets, and insufficient consumption of fruits, vegetables, and whole grains are dietary patterns that have been closely linked to insulin resistance, oxidative stress, and chronic inflammation—all of which are hallmarks of PCOS (8). These findings lend credence to the theory that dietary practices play a major role in the pathophysiology of PCOS by modifying molecular pathways related to steroidogenesis, insulin signaling, and inflammatory regulation (9–11).

Micronutrients, vitamins and minerals, are vital cofactors for many biochemical processes that control hormone synthesis, energy metabolism, antioxidant defense, and cell signaling. Minerals aid in enzymatic activity, insulin receptor function, redox balance, and steroidogenic regulation, whereas vitamins mainly serve as antioxidants and enzymatic cofactors. Precision nutrition strategies in PCOS require a focused understanding of the roles of vitamins and minerals (3, 12).

Current research (13, 14) demonstrates that the status of micronutrients in the body significantly affects how PCOS develops and responds to treatment. Deficiencies of vitamin D alongside magnesium, zinc, selenium, B-vitamins and antioxidant vitamins aggravate insulin resistance, inflammation, oxidative stress and reproductive dysfunction (10). Correcting these deficiencies can improve metabolic, hormonal and inflammatory profiles by modulating molecular pathways like PI3K/Akt, NF-κB, Nrf2, AMPK, mTOR, MAPK/ERK, and steroidogenic enzyme activity.

Furthermore, women with PCOS are frequently deficient in these essential nutrients (15), contributing to disruptions in folliculogenesis, steroidogenesis, methylation pathways, and mitochondrial function, which in turn maintain anovulation, hyperandrogenism, and metabolic disturbances (3).

Given the growing body of evidence linking micronutrient status to PCOS pathophysiology, there is a need to consolidate existing mechanistic and clinical evidence. This review aims to provide a molecular level understanding of how key micronutrients affect PCOS pathways, highlight gaps in current knowledge and explore their therapeutic potential as adjuncts to conventional treatment. A mechanistic understanding of nutrient-pathway interactions provides the scientific basis for using micronutrient therapy as a non-pharmacological adjunct.

Beyond its effects on reproduction, PCOS is a significant public health issue with extensive metabolic, psychological, and socioeconomic ramifications. Type 2 diabetes mellitus, cardiovascular disease, non-alcoholic fatty liver disease, infertility, anxiety, and depression are all substantially more common in women with PCOS. Across all socioeconomic levels, these comorbidities have a disproportionate impact on long-term healthcare costs, productivity, and quality of life. Crucially, PCOS affects women in both urban and rural areas, highlighting the fact that it is a systemic condition influenced by biological susceptibility interacting with environmental and nutritional factors rather than a lifestyle disorder exclusive to particular social groups (16, 17).

The following sections examine each micronutrient’s molecular targets and clinical relevance, integrating evidence from observational studies and randomized trials with mechanistic data from cellular and animal models to clarify their potential to modulate key pathways underlying insulin resistance, hyperandrogenism, oxidative stress, and inflammation in PCOS.

## Micronutrients in PCOS and their molecular mechanisms

Nutritional deficiencies are frequently observed in women with PCOS and may contribute to the metabolic and reproductive abnormalities associated with the disorder. Observational studies have consistently shown low levels of vitamin D, magnesium, zinc, selenium, B vitamins and calcium in PCOS which are associated with insulin resistance, inflammation, oxidative stress, irregular periods and cardiovascular risk. These deficiencies are not just coincidental but can exacerbate molecular dysregulation such as impaired insulin signaling, altered steroidogenesis and redox imbalance (18–20). Given these consistent findings, it becomes essential to examine the role of individual vitamins and minerals in PCOS, as each micronutrient contributes uniquely to the regulation of metabolic, hormonal, and inflammatory pathways. To avoid redundancy, common signaling pathways such as PI3K/Akt, NF-κB, and Nrf2 are discussed within nutrient-specific contexts rather than repeated in full detail across sections.

Vitamins are vital organic compounds that aid in hormone synthesis, cellular signaling, antioxidant defense, and enzyme activity. Vitamin A, vitamin C, vitamin D, vitamin E, and B-complex vitamins (B1, B2, B3, B5, B6, B7, B9/folate, B12) are the essential vitamins crucial for good health. Inadequate consumption of these vitamins can affect the hormonal, inflammatory, and metabolic abnormalities frequently observed in PCOS.

## Vitamin D

Strong and consistent evidence connecting vitamin D deficiency to both metabolic and reproductive dysfunction in PCOS calls for a separate, in-depth discussion. In contrast to other micronutrients, it functions as a secosteroid hormone that regulates gene transcription through the vitamin D receptor, affecting follicular development, immunological response, ovarian steroidogenesis, and insulin sensitivity. Its deficiency is also one of the most common micronutrient abnormalities in PCOS worldwide, and its therapeutic significance is supported by a wealth of clinical and mechanistic data.

The fat-soluble secosteroid vitamin D functions as a prohormone, with its active form (calcitriol) acting as a hormone that regulates calcium–phosphate homeostasis, reproductive function, metabolic processes, and immune system activity. Vitamin D deficiency among women with PCOS is linked to insulin resistance alongside chronic inflammation and hyperandrogenism as well as ovulatory dysfunction. Through ligand-dependent activation of the vitamin D receptor (VDR), vitamin D regulates gene transcription, thereby influencing key biological pathways such as folliculogenesis, steroidogenesis, and insulin receptor signaling. These mechanistic insights align with clinical findings, where supplementation has been shown to improve insulin sensitivity, reduce testosterone levels, and normalize menstrual cycles, supporting its role as a promising adjunct treatment for PCOS. (4, 21).

Vitamin D is essential to the process of folliculogenesis due to its influence on steroid hormone production. In humans, calcitriol was shown to influence the activity of 3β-hydroxysteroid dehydrogenase (3β-HSD, an enzyme crucial for the production of progesterone and other steroid hormones) in granulosa cells, suggesting that vitamin D increases progesterone synthesis. The vitamin D receptor (VDR) exerts its genomic effects by forming a ligand-activated transcriptional complex that binds to vitamin D–responsive elements (VDREs) in target genes, thereby directly regulating the expression of steroidogenic enzymes (22).

Vitamin D or its active form 1,25-dihydroxyvitamin D_3_ (calcitriol) has been shown to have an influence on the expression of AMH (anti-Müllerian hormone, a hormone that regulates follicle development by inhibiting premature follicle recruitment) and its receptor (AMHR2) in granulosa cells. For instance, a study demonstrated that vitamin D3 treatment reduced AMHR2 expression in cumulus granulosa cells indicating a possible modulatory affect on AMH signaling pathways important for follicular development and maturation. If vitamin D3 creates a functional environment to lessen the inhibitory effects of AMH on folliculogenesis, follicular maturation could occur in patients with PCOS (21, 23).

Deficiency in vitamin D affects the grading of ovarian granulosa cells in terms of proliferation and apoptosis through micro-RNA changes. In particular, vitamin D deficiency was identified to negatively regulate microRNA-196b- 5p (regulatory microRNA that helps maintain normal granulosa cell growth and survival) which negatively affected its target genes operating in granulosa cells. This disruption could potentially lead to the attenuated follicular development that is characteristic of PCOS (24).

Insulin resistance is a common metabolic abnormality in women with polycystic ovary syndrome (PCOS). The vitamin D metabolite has been suggested to improve insulin sensitivity through modulation of insulin receptor gene expression. While direct evidence in women with PCOS is limited, research has shown that vitamin D can enhance insulin receptor expression and insulin mediated glucose transport in many tissues, potentially improving insulin sensitivity in patients with PCOS (25).

Chronic low-grade inflammation and oxidative stress are defining characteristics of PCOS. Vitamin D has been shown to have anti-inflammatory effects via downregulation of pro-inflammatory cytokines, such as TNF-α and interleukins. One report of a randomized controlled trial that allocated supplementation with vitamin D to infertile women with PCOS found that supplementation markedly reduced high-sensitivity C-reactive protein (hs-CRP) levels and increased total antioxidant capacity, indicating the potential of vitamin D to mitigate inflammation and oxidative stress in PCOS (26).

**Figure 2:**
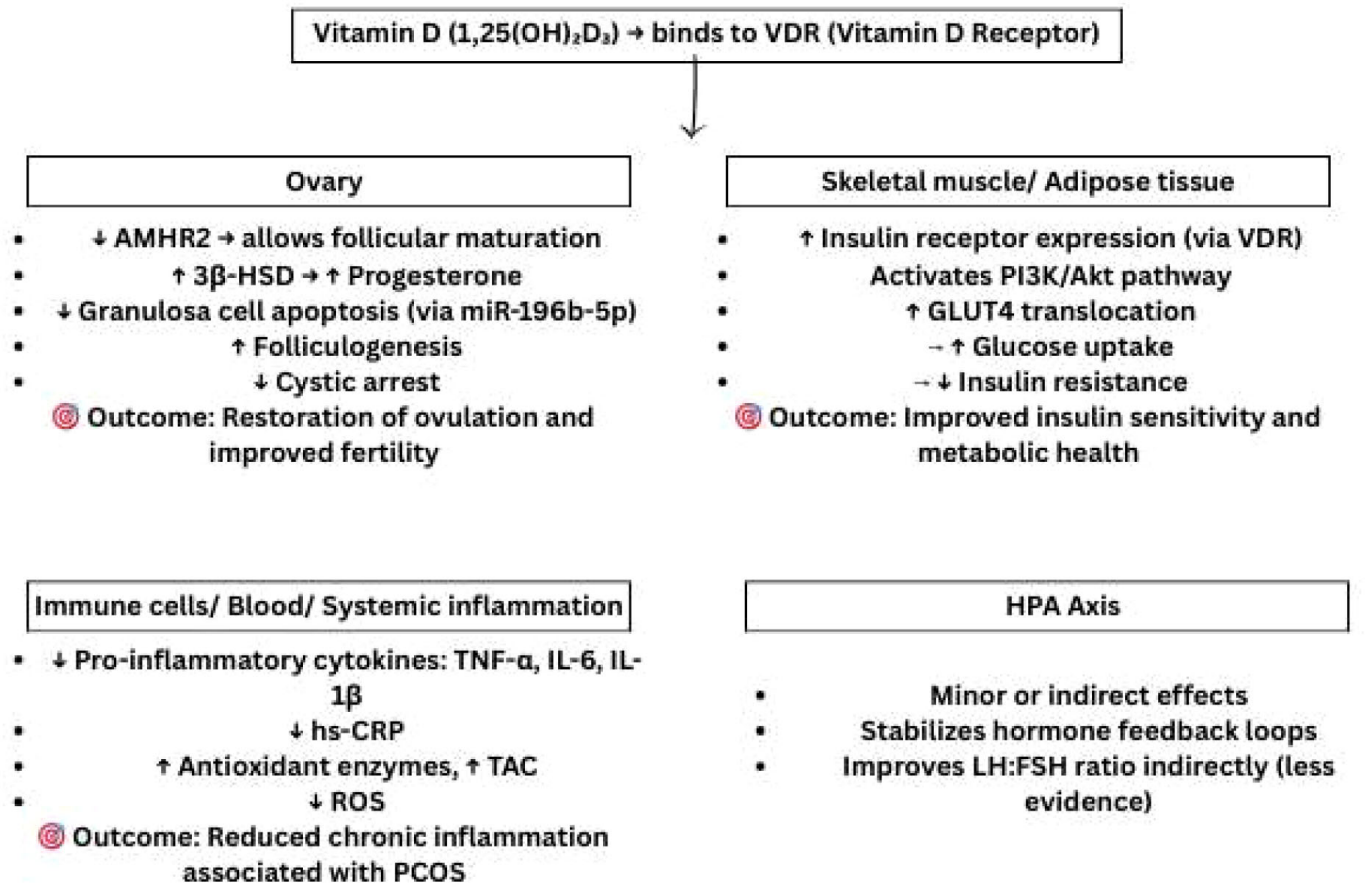
Organ-wise molecular mechanisms of vitamin D in mitigating PCOS pathology through modulation of ovarian folliculogenesis, insulin sensitivity, and systemic inflammation. VDR, Vitamin D Receptor; AMHR2, Anti-Müllerian Hormone Receptor 2; 3β-HSD, 3β-Hydroxysteroid Dehydrogenase; miR, microRNA; GLUT4, Glucose Transporter Type 4; PI3K, Phosphoinositide 3-Kinase; Akt, Protein Kinase B; TNF-α, Tumor Necrosis Factor-alpha; IL, Interleukin; hs-CRP, High-sensitivity C-Reactive Protein; TAC, Total Antioxidant Capacity; ROS, Reactive Oxygen Species; HPA, Hypothalamic–Pituitary–Adrenal; LH, Luteinizing Hormone; FSH, Follicle-Stimulating Hormone. Symbols: ↑, Increased; High, ↓, Decreased/Low.

These findings also laid emphasis on how vitamin D influences multiple molecular aspects involved with the pathogenesis of PCOS. It is hence crucial to maintain a good level of vitamin D as it plays a role in the management of both the reproductive and metabolic disorders related to PCOS (**Figure 2**).

## Vitamin B-complex

**Figure 3:**
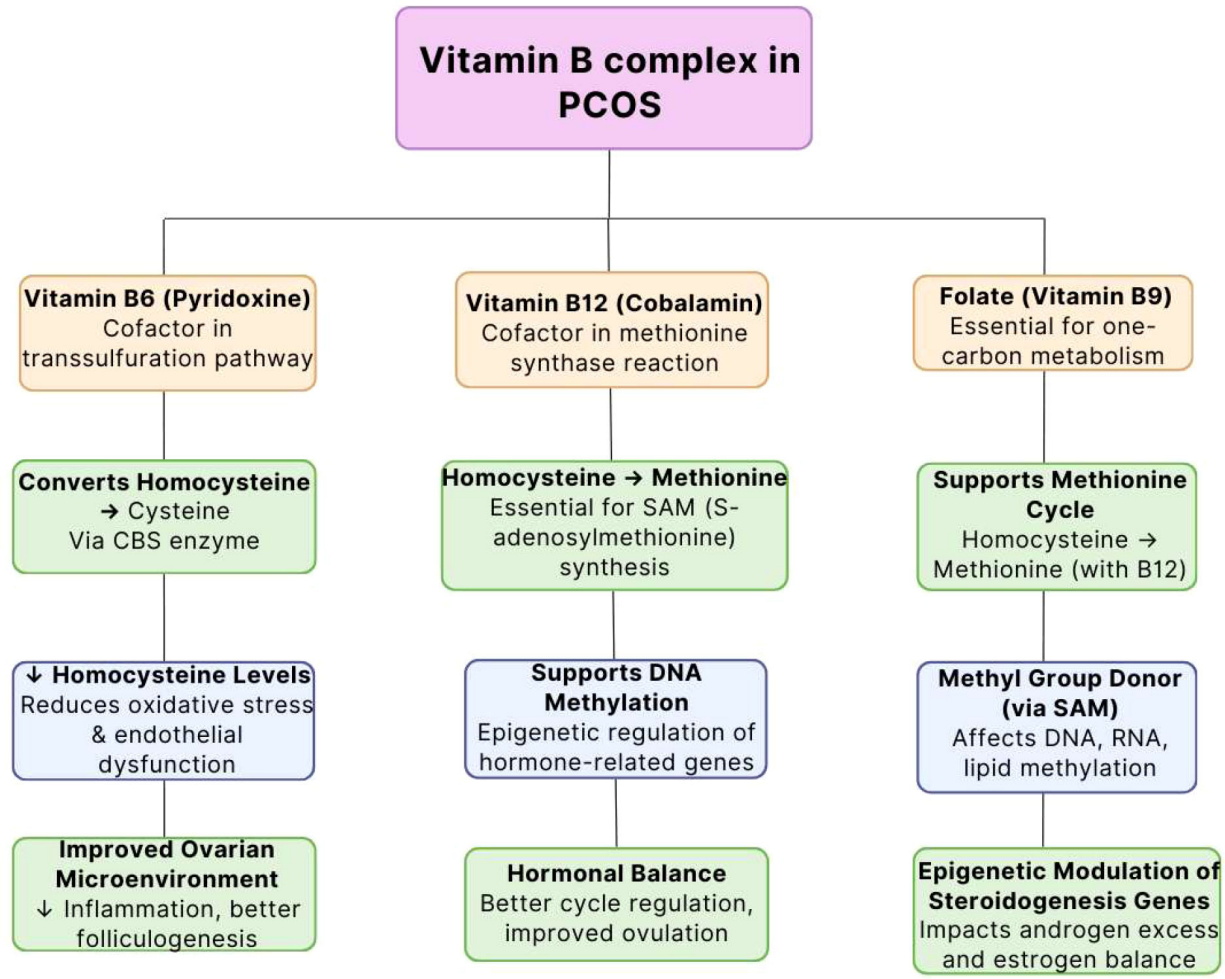
Functions of B-vitamins in PCOS: methylation, homocysteine metabolism, and hormonal regulation. Symbols: ↑, Increased; High, ↓, Decreased/Low.

The intricate roles of B-complex vitamins, particularly B6, B12, and folate, involve their function as essential cofactors in processes such as one-carbon metabolism and DNA methylation alongside neurotransmitter synthesis and homocysteine regulation. Women with PCOS often show elevated homocysteine levels which connect to cardiovascular risk factors along with insulin resistance and dysfunctional ovarian activity. These metabolic disturbances become more severe when B- complex vitamin deficiencies disrupt both methylation processes and redox balance. Studies demonstrate that B-complex vitamin supplementation lowers homocysteine levels while enhancing ovulatory function and insulin sensitivity which indicates these nutrients might serve as potential therapeutic agents for PCOS (27) (**Figure 3**).

Homocysteine is formed in the metabolic pathway associated with methionine and its conversion to cysteine and methylation requires vitamin B6, vitamin B12, and folic acid. Lack in these vitamins will affect these pathways thus resulting to hyperhomocysteinemia which is related to cardiovascular diseases, bone metabolic complications as well as neuropathies. In particular, plasma total homocysteine (Hcy) concentrations are known to be increased in women with PCOS. A meta-analysis of 75 studies showed that the circulating homocysteine levels (SMD: 0.82; 95% Confidence Interval [CI]: 0.62–1.02; p < 0.001) of control group was significantly lower than the PCOS group. This elevation was evident in the following subgroups: world region, assay method and insulin resistance status (28).

Intervention studies have shown that B-complex vitamin supplements may lower homocysteine levels in women who have PCOS. A randomized trial found that patients taking metformin along with B-group vitamins (B1, B6, and B12 included) saw their homocysteine levels drop by 21.17% over 12 weeks. In contrast, those on metformin alone experienced a 26.5% increase (29, 30).

Research also indicates that folic acid supplements can reduce homocysteine concentrations in women with PCOS who have high homocysteine levels. This holds true whether they’re insulin resistant or not. After taking folic acid for 3 months, both insulin-resistant and non-insulin-resistant groups showed significant decreases in their homocysteine levels (31).

Folate and vitamin B12 play key roles in one-carbon metabolism. This process is key to DNA synthesis and methylation. Folate derivatives help make purines and pyrimidines. They also help turn homocysteine into methionine—a step that needs vitamin B12 too. This process is key to methylate DNA, RNA, proteins, and lipids. As a result, it affects gene expression and how cells work. In PCOS unusual DNA methylation patterns may cause the syndrome. These patterns affect genes that control hormones and metabolism. By having enough folate and vitamin B12, we might change these epigenetic shifts. This could lead to better hormone balance and metabolism in PCOS patients (32).

The relationship link between high homocysteine levels and PCOS, as well as the positive outcomes of B-complex vitamin supplementation on homocysteine metabolism, indicate that these vitamins might offer useful therapeutic value in managing PCOS. While these vitamins seem to help improve biochemical markers PCOS-related measurements, further investigation is required to understand the clinical impacts such as ovulation, fertility, and metabolic health.

## Vitamin E and C: antioxidant defense mechanism via the Nrf2 pathway and oxidative stress reduction in ovarian tissue

The dietary antioxidant Vitamins E (α-tocopherol) and C (ascorbic acid) exhibit powerful protective functions against cellular oxidative damage while simultaneously regulating inflammatory responses. The pathogenesis of PCOS involves oxidative stress as a fundamental factor which disrupts follicular development while inducing granulosa cell apoptosis and promoting insulin resistance (1). Animal and human studies of PCOS demonstrate that combined vitamin E and C supplementation produces synergistic effects to decrease oxidative damage while enhancing ovarian function (33).

Nrf2 is a master transcription factor that plays a key role in regulating the expression of antioxidant proteins that protect the body from oxidative damage and inflammation. In women with PCOS, lower levels of Nrf2 were observed in those with hyperandrogenism, insulin resistance and obesity. Vitamins E and C are powerful antioxidants, helping to mitigate the actions of oxidative stress (OS) in ovarian tissue. Research showed that these vitamins reduced OS-induced apoptosis by stimulating Nrf2 signaling, lowering intracellular reactive oxygen species (ROS) levels, and inhibiting caspase activity. Interestingly, the knockdown of Nrf2 inhibited the cytoprotective effects of vitamin E on granulosa cells. This demonstrates that Nrf2 is critically involved in mediating the antioxidant effects of both vitamins E and C on ovary cells (33–35).

Vitamin E, specifically α-tocopherol, is a fat-soluble antioxidant that can prevent tissues from lipid peroxidation. Vitamin E has been shown to selectively upregulate the Nrf2-mediated defense system through the PI3K/AKT and ERK1/2 pathways in bovine granulosa cells to enhance cell proliferation and prevent apoptosis. Overall, the findings suggest vitamin E may enhance ovarian function by reducing oxidative stress through Nrf2 activation (36, 37).

Vitamin C is an antioxidant that is water soluble, therefore a vital cofactor in collagen synthesis, vascular development and cell proliferation. Vitamin C reduces oxidative stress damage in PCOS, through upregulating antioxidant enzyme expression. Vitamin C supplementation in mice increased total antioxidant capacity and decreased damage to oxidative stress in ovarian tissues, suggesting a potential therapeutic role in the management of PCOS (36).

**Figure 4:**
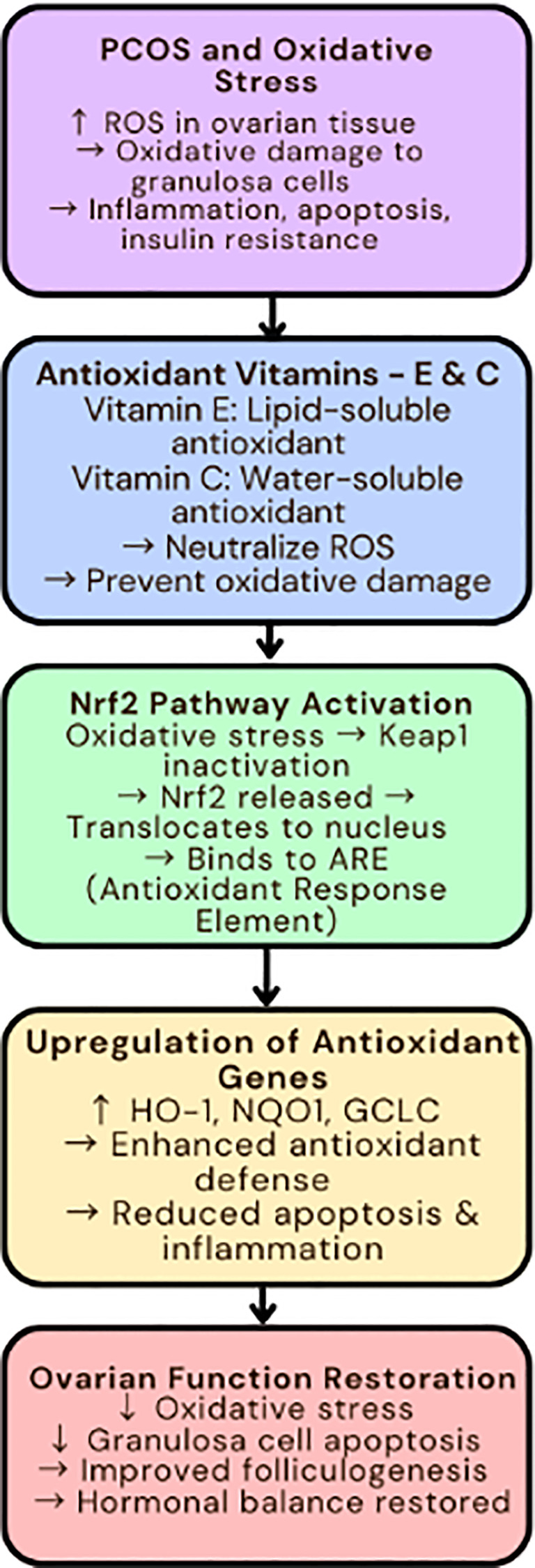
Antioxidant vitamins and Nrf2 pathway in oxidative stress-induced PCOS. ROS, Reactive Oxygen Species; Nrf2, Nuclear Factor Erythroid 2–Related Factor 2; Keap1, Kelch-like ECH-associated Protein 1; ARE, Antioxidant Response Element; HO-1, Heme Oxygenase 1; NQO1, NAD(P)H Quinone Dehydrogenase 1; GCLC, Glutamate-Cysteine Ligase Catalytic Subunit; PCOS, Polycystic Ovary Syndrome. Symbols: ↑, Increased; High, ↓, Decreased/Low.

Administration of vitamins E and C together has been described as having synergistic effects on oxidative stress. This research showed that the combination therapy of vitamins E and C reduced the effects of oxidative stress-induced apoptosis in granulosa cells showing that it was activating Nrf2 signaling, lowering ROS levels intracellularly, and inhibiting caspase activity. These synergistic effects highlight the possibilities of combined antioxidant therapy in oxidative stress-induced ovarian dysfunction (33) (**Figure 4**).

Minerals are essential inorganic elements that support cellular and systemic health through their structural, enzymatic, and signaling roles. Among the essential minerals are calcium, iron, iodine, potassium, sodium, phosphorus, magnesium, zinc, selenium, and chromium.

## Magnesium: enhancing insulin sensitivity and GLUT4 activation

**Figure 5:**
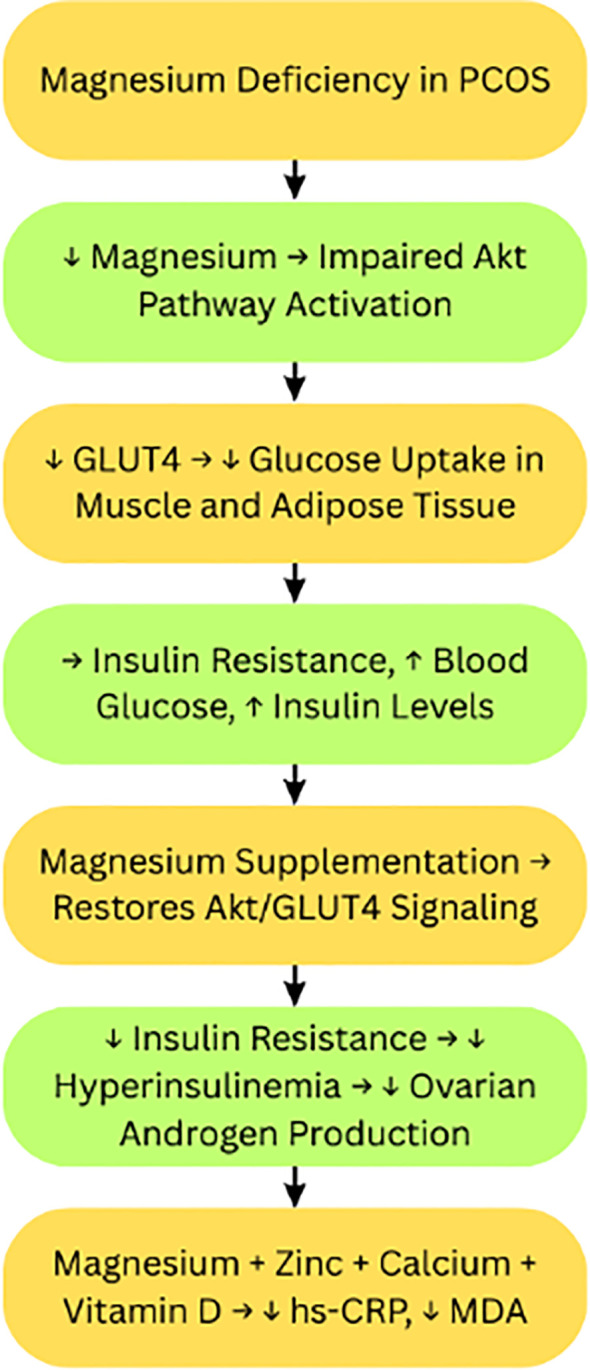
Magnesium deficiency and supplementation effects in PCOS. PCOS, Polycystic Ovary Syndrome; Akt, Protein Kinase B; GLUT4, Glucose Transporter Type 4; hs-CRP, High-sensitivity C-Reactive Protein; MDA, Malondialdehyde. Symbols: ↑, Increased; High, ↓, Decreased/Low.

Magnesium is an essential intracellular mineral, playing roles in over 300 enzymatic reactions, especially those related to glucose metabolism, insulin signaling, and cellular energy production. Women with PCOS often show decreased serum magnesium levels, which correlate with insulin resistance, increased inflammation, and metabolic dysfunction. Magnesium promotes phosphorylation of Akt and stimulates the translocation of GLUT4 (glucose transporter type 4), thereby enhancing insulin sensitivity and glucose uptake. Supplementation was shown to improve insulin resistance and oxidative stress, along with clinical manifestations like hirsutism in PCOS subjects (37) (**Figure 5**).

Magnesium is critical to glucose metabolism and insulin signaling. In studies of adipocytes with magnesium deficiency, insulin-dependent glucose uptake has been shown to be reduced by 50%. This impaired glucose uptake was associated with reduced phosphorylated Akt activation and translocation of GLUT4 to the plasma membrane, although the phosphorylation of the insulin receptor was unchanged. These results indicate that magnesium is necessary for post-insulin receptor activation effects involving GLUT4-mediated uptake of glucose (37).

Additional research focusing on type 2 diabetic rat models, consisting of magnesium supplementation, showed that magnesium supplementation increases the protein expression of insulin receptors and GLUT4 in skeletal muscle and adipose tissue primarily through improved insulin-signaling efficiency and enhanced activation of downstream pathways such as PI3K/Akt. This increase of insulin receptors and GLUT4 proteins subsequently led to improved glucose tolerance, increased insulin sensitivity, and noticeably reduced markers of oxidative stress, as observed by low malondialdehyde (MDA) levels (biomarker for oxidative stress) (38).

In females with PCOS, magnesium supplementation is associated with decreased oxidative stress and inflammation when combined with zinc, calcium, and vitamin D, owing to decreased levels of hs-CRP and MDA in addition to decreased levels of insulin resistance indices (39).

## Calcium: insulin signaling, ovarian function, and metabolic homeostasis

A vital intracellular second messenger, calcium is involved in the synthesis of steroid hormones, follicular maturation, and insulin secretion. Insulin resistance and metabolic dysfunctions frequently seen in PCOS have been linked to disruptions in calcium homeostasis. The interdependence of these micronutrients is shown by clinical investigations showing changed serum calcium levels in women with PCOS, frequently in conjunction with vitamin D insufficiency. By modifying intracellular signaling cascades and mitochondrial activity, calcium affects insulin-mediated glucose absorption. It also plays a role in oocyte maturation and ovulatory processes. Research indicates that supplementing with both calcium and vitamin D improves inflammatory markers, insulin sensitivity, and menstrual regularity in women with PCOS, supporting its inclusion as a physiologically significant micronutrient (40).

## Zinc: modulating inflammation, androgen production, and insulin resistance

Zinc is an important trace mineral that is involved in many different physiological functions, including insulin production, oxidative stress responses, and regulating reproductive hormones. In women with PCOS serum zinc levels are decreased, which is related to hyperinsulinemia, inflammation, and dyslipidemia. Zinc also affects antioxidant-enzyme activity (e.g., SOD- superoxide dismutase, catalase), 5α-reductase (that limits the conversion of testosterone to DHT - dihydrotestosterone), and mTOR expression, all of which are related to the pathology of PCOS. There is a plausible rationale for zinc as supplementation to improve insulin resistance, lipid metabolism and hyperandrogenism in PCOS animals and patients (41).

**Figure 6:**
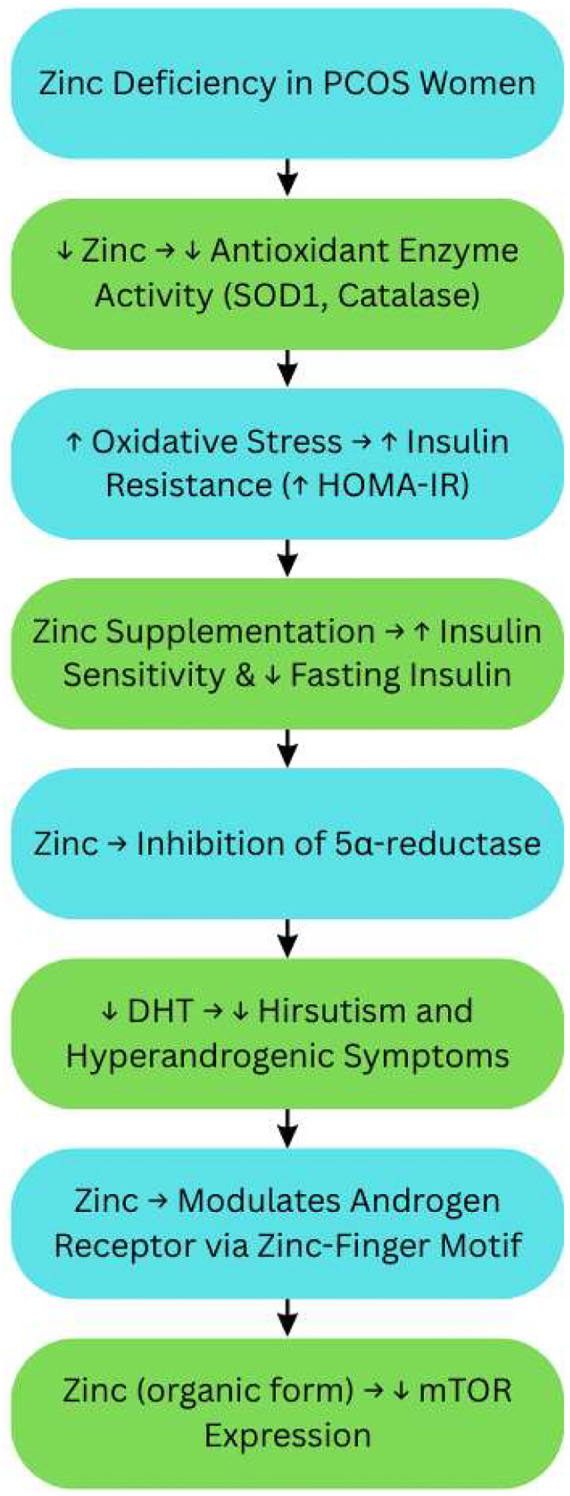
Zinc’s role in antioxidant defense and androgen regulation in PCOS. PCOS, Polycystic Ovary Syndrome; SOD1, Superoxide Dismutase 1; HOMA-IR, Homeostatic Model Assessment of Insulin Resistance; DHT, Dihydrotestosterone; mTOR, Mammalian Target of Rapamycin. Symbols: ↑, Increased; High, ↓, Decreased/Low.

Zinc is necessary for many physiological functions including insulin production, storage, and action. In women with PCOS, serum zinc is frequently decreased, and inversely correlated with insulin resistance and oxidative stress. Zinc supplementation has been shown to decrease insulin levels and significantly improve the homeostasis model assessment of insulin resistance (HOMA-IR) index to evaluate insulin sensitivity (41) (**Figure 6**).

Zinc, as a cofactor for the antioxidant enzymes such as superoxide dismutase (SOD1) and catalase (CAT), may be helpful in reducing oxidative stress from insulin resistance. Zinc supplementation in women with PCOS and hyperlipidemia resulted in a decrease in MDA as a marker of oxidative stress along with improved lipid levels (42).

Zinc also has an influence on androgen metabolism. Zinc has been shown to inhibit 5α-reductase, which converts testosterone to its more biologically active form, DHT, which may lower some of the hyperandrogenism signs and symptoms, e.g., hirsutism. Zinc also influences androgenic receptors, which are characterized by zinc finger motifs, suggesting zinc status may modulate androgen receptor activity (MDPI) (41).

In animal models of PCOS, organic zinc supplementation has been associated with downregulation of the mammalian target of rapamycin (mTOR) gene regarding expression which is a critical modulator of cell growth and metabolism. The downregulation of mTOR was associated with less insulin resistance, lower LH and testosterone levels, and improved ovarian morphology (42).

## Selenium: selenoproteins in oxidative stress and thyroid function

**Figure 7:**
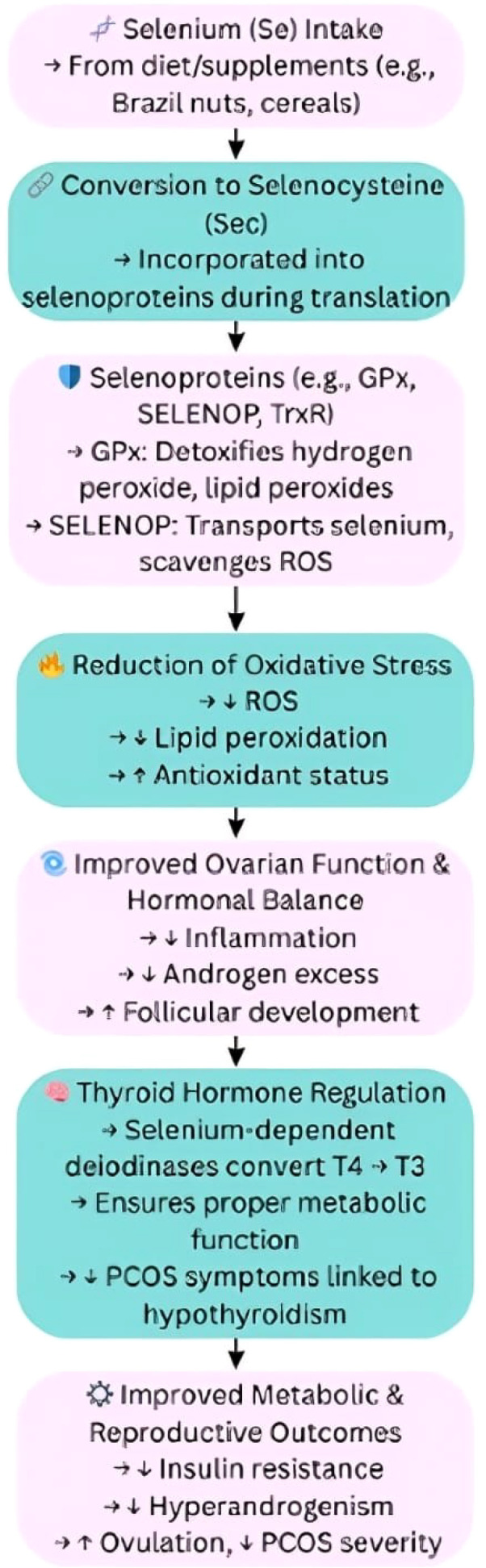
Selenium’s influence on antioxidant activity, thyroid function, and PCOS outcomes. Se, Selenium; GPx, Glutathione Peroxidase; SELENOP, Selenoprotein P; TrxR, Thioredoxin Reductase; ROS, Reactive Oxygen Species; T4, Thyroxine; T3, Triiodothyronine; PCOS, Polycystic Ovary Syndrome. Symbols: ↑, Increased; High, ↓, Decreased/Low.

Selenium is a trace element incorporated in the antioxidant selenoproteins such as glutathione peroxidase and selenoprotein P (SELENOP) that neutralize oxidative stress and maintain redox balance. Selenium status has also been inversely correlated in PCOS with insulin resistance and lipid peroxidation, further supporting a role in metabolic protection. Beyond its antioxidant role, selenium also supports thyroid hormone synthesis and mitochondrial function, two processes that are commonly disrupted in PCOS. As demonstrated by studies, selenium supplementation seems to aid insulin sensitivity and the antioxidant state in PCOS women, hence suggesting its potential application in treatment (43) (**Figure 7**).

Selenoproteins reduce oxidative stress by detoxifying reactive oxygen species (ROS), which are elevated in PCOS and have a role in its pathophysiology. In a cross-sectional study with 125 women with PCOS, elevated serum selenium and SELENOP were shown to have higher total antioxidant capacity and GPx activity (selenium-dependent antioxidant enzyme) as well as lower markers of lipid peroxidation. These results suggest potential benefit for selenium supplementation in order to enhance antioxidant defenses in female patients with PCOS (43).

Selenium is critical in the synthesis and metabolism of thyroid hormones. Selenium is a key cofactor for the enzymes iodothyronine deiodinases and thyroid peroxidase (TPO). The two key roles of these enzymes are to activate and regulate thyroid hormones, which regulate metabolic functions in the body. Thyroid dysfunction in PCOS is a common occurrence and can lead to metabolic disturbance, so ensuring adequate selenium levels may assist in the meeting of thyroid hormone needs and help mitigate some symptoms of PCOS (44).

Mitochondria are essential for energy production and are also a major source of ROS. Because mitochondria produce selenium containing enzymes that can help maintain redox balance necessary for cellular function, selenium is important in thyroid disorders that can happen in conjunction with PCOS. Selenium plays a significant role in the maintenance of mitochondrial function. Selenium could possibly help reduce the metabolic complications of PCOS by promoting better energy metabolism and decreasing oxidative stress (44).

## Chromium: insulin potentiation and metabolic regulation

**Figure 8:**
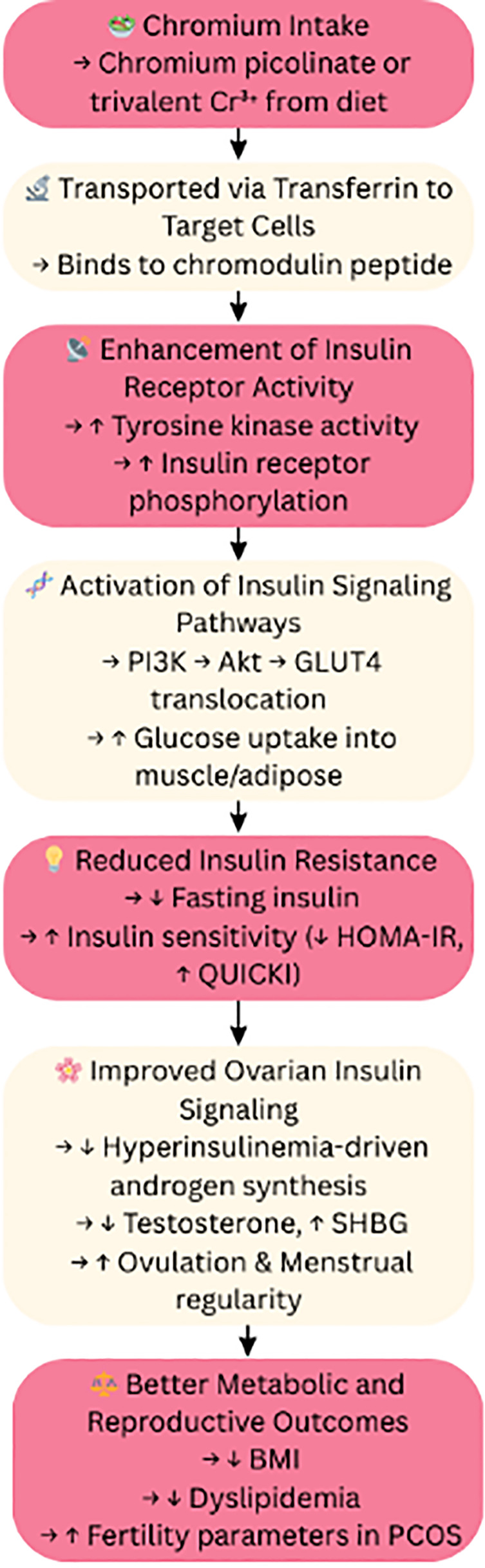
Chromium’s role: insulin and metabolic regulation. Cr³^+^, Trivalent Chromium; PI3K, Phosphoinositide 3-Kinase; Akt, Protein Kinase B; GLUT4, Glucose Transporter Type 4; HOMA-IR, Homeostatic Model Assessment of Insulin Resistance; QUICKI, Quantitative Insulin Sensitivity Check Index; SHBG – Sex Hormone Binding Globulin; BMI, Body Mass Index; PCOS, Polycystic Ovary Syndrome. Symbols: ↑, Increased; High, ↓, Decreased/Low.

The chromium element, especially the trivalent variant chromium picolinate, plays an important role in augmenting the action of insulin by stimulating receptor activity. In PCOS, chromium therapy has recorded effects on insulin by lowering fasting levels of the hormone, improving HOMA-IR scores, as well as regularizing menses. It can also benefit ovulatory function and reduce androgen excess, but the exact mechanism is still unclear. This dual influence on metabolic and reproductive axes creates an interesting possibility for incorporating chromium in the holistic treatment of PCOS (45, 46) (**Figure 8**).

A randomized, double-blind, placebo-controlled trial of 64 women with PCOS assessed the effectiveness of chromium supplementation in the management of PCOS. Following supplementation with chromium, significantly reduced fasting insulin values were found in the chromium group and HOMA-IR and QUICKI (Insulin sensitivity test) scores were improved. These findings suggest that chromium supplementation may be beneficial in managing insulin resistance in PCOS patients (45).

Besides metabolic outcomes, chromium supplementation has also been tied to beneficial reproductive outcomes in PCOS. In a study with 85 women, after 6 months of chromium picolinate supplementation, they exhibited substantial reductions in fasting insulin and BMI, as well as an increase in ovulation and menstrual cycle regularity. These data suggest that chromium may help restore female reproductive function in cases of PCOS (46).

Mechanistically, chromium is thought to enhance insulin receptor activity and increase insulin receptor quantity, in addition, promote the activation of downstream signaling pathways involved in glucose metabolism. Collectively, these actions could enhance insulin sensitivity and glucose uptake, addressing one of the more original metabolic derangements in PCOS. Additional research is still needed to focus on these mechanisms, but the evidence currently available supports the notion that chromium has potential as an adjunct therapy in the management of PCOS (47).

Despite conflicting results, chromium is still discussed in relation to PCOS because a number of studies show improvements in menstrual cyclicity and insulin sensitivity, which may be advantageous for some individuals. However, different chromium formulations, dosages, treatment durations, baseline metabolic statuses, and cohort characteristics are probably responsible for the variable findings across trials. Limited long-term safety data further argue against routine use. Thus, its inclusion emphasizes the necessity of identifying responder phenotypes and mechanistic markers that may shed light on its therapeutic value in PCOS.

Building on the role of each vitamin and mineral, it is clear that targeted nutritional strategies offer meaningful therapeutic potential in PCOS management. Evidence from randomized controlled trials show that targeted supplementation alone or in combination can improve insulin sensitivity, reduce inflammatory markers and support reproductive outcomes. Notably multi nutrient interventions (e.g. magnesium, zinc, calcium and vitamin D co-supplementation) have shown synergistic benefits, hence we need to focus on integrative nutritional approaches. Future studies should focus on individualized supplementation based on PCOS phenotype, genetic background and nutrient-nutrient interactions (48) (**Table 1**).

**Table 1:** Common nutrient deficiencies and their clinical manifestations in PCOS.

Nutrient	Deficiency prevalence in PCOS	Associated manifestations
Vitamin D	High	Insulin resistance, obesity, dyslipidemia, reproductive dysfunction
Magnesium	Moderate to High	Insulin resistance, inflammation, hirsutism
Zinc	Moderate	Hyperinsulinemia, inflammation, dyslipidemia
Selenium	Variable	Oxidative stress, insulin resistance
B-Vitamins	Moderate	Elevated homocysteine, cardiovascular risk
Calcium	Variable	Insulin resistance, menstrual irregularities

A structured summary that differentiates nutrients supported by clinical trials from those largely supported by mechanistic or experimental data has been provided to give a clearer picture of the evidence landscape around micronutrient research in PCOS (**Table 2**).

**Table 2:** Summary of clinical and experimental evidence supporting micronutrient involvement in PCOS.

Micronutrient	Type of evidence	Key observations in PCOS	Representative evidence
Vitamin D	Strong clinical + mechanistic	Improved insulin sensitivity, reduced androgen levels, improved ovulatory function	RCTs (49), meta-analyses (50), granulosa cell studies (51)
B-complex vitamins (Folate, B6, B12)	Moderate clinical + mechanistic	Reduced homocysteine, improved methylation status, cardiometabolic risk modulation	RCTs (29), meta-analyses (28)
Magnesium	Moderate clinical + mechanistic	Improved insulin resistance, reduced inflammation, enhanced GLUT4 activity	RCTs (52), animal and cellular studies (53)
Zinc	Moderate clinical + experimental	Reduced oxidative stress, modulation of androgen metabolism, improved lipid profile	RCTs (41), animal studies (45)
Selenium	Limited clinical + strong experimental	Improved antioxidant capacity, mitochondrial and thyroid-related metabolic regulation	Observational studies, mechanistic studies (54)
Calcium	Emerging clinical evidence	Improved insulin sensitivity and menstrual regularity when combined with vitamin D	Small clinical trials (55)
Chromium	Conflicting clinical + experimental	Improved insulin sensitivity in some studies; inconsistent reproductive outcomes	RCTs with variable findings (56)

## Discussion

Micronutrients play a significant role in the hormonal, metabolic, and inflammatory issues associated with PCOS. This review brings together molecular and clinical findings to show how vitamins and minerals affect insulin signaling, steroid hormone production, redox balance, and inflammatory pathways such as PI3K/Akt, NF-κB, Nrf2, and AMPK. However, despite strong preclinical evidence, turning micronutrient therapy into standard clinical guidelines is complicated. There are ongoing scientific debates, contradictory mechanisms, and methodological challenges that need further exploration.

### Different schools of thought and ongoing controversies

One major issue is whether micronutrient deficiencies in PCOS cause, result from, or may aggravate metabolic imbalances. Some researchers claim that deficiencies, especially in vitamin D, magnesium, zinc, and B vitamins, directly hinder insulin receptor signaling and ovarian hormone production, contributing to PCOS development. Others argue these deficiencies stem from obesity, chronic inflammation, and poor dietary habits associated with PCOS, suggesting that supplements mainly correct secondary nutritional issues rather than address the disease mechanisms.

Controversy also surrounds vitamin D supplementation. While several trials report improvements in insulin sensitivity, inflammation, and follicular growth, other studies see minimal or inconsistent results. These differences have led to diverse opinions on whether vitamin D acts as a prohormone that directly impacts ovarian signaling, or if its benefits mainly come from improved metabolic health linked to higher vitamin D levels.

The significance of chromium, selenium, and zinc supplementation is another topic of debate. Some experts support their regular use in clinical settings due to their positive effects on insulin function, antioxidant protection, and androgen levels. Others warn against universal supplementation because of varying treatment responses, safety concerns over long-term use, and mixed outcomes in randomized controlled trials.

### Challenges and research gaps

#### Bioavailability issues

The effectiveness of nutritional supplements for use in managing PCOS is frequently undermined by bioavailability concerns. Factors such as nutrient-nutrient interactions, absorption capacities by individuals, or the form of the supplement can greatly influence nutrient bioavailability. For example, some mineral absorption (e.g. magnesium) can be adversely influenced by other dietary components, which can result in reduced therapeutic efficacy. Furthermore, with lack of standardizations in formulation and dosages across studies, makes it difficult to know the effective dose when considering efficacy of supplements. These complications signal the need to conduct more research regarding optimizing formulations and bioavailability to help women with PCOS to better manage their conditions (57, 58).

#### Individual variability and nutrigenomics

Responses to nutritional interventions in PCOS are individualized reflecting their genetic variably where variances in genetic polymorphisms can affect nutrient metabolism, absorption and action which can all lead to differential responses in individuals. For instance, polymorphisms in genes regulating insulin signaling pathways may influence how effectively dietary interventions improve insulin sensitivity. Therefore, accounting for genetic variability is essential when developing nutrient-specific strategies to manage PCOS symptoms. Unfortunately, there is very little research in this field, and we need more studies that describe the complex relationship between genetics and nutrition in PCOS (59).

#### Need for large-scale randomized controlled trials

Despite the fact that a large number of studies have examined nutritional interventions in PCOS, many of them are limited by methodological irregularities, short follow-up periods, and small sample sizes. These constraints cause a disconnect between research findings and clinical practice and impede the creation of strong clinical guidelines for nutritional management. Large-scale, carefully planned randomized controlled trials (RCTs) that assess the safety and effectiveness of dietary interventions for PCOS are in dire need. To strengthen the body of evidence supporting clinical application, such studies should include a variety of populations, standardize intervention protocols, and offer longer-term follow-up (60, 61).

### Therapeutic implications: integrating micronutrients with pharmacological and complementary approaches

Pharmacological treatments include insulin sensitisers, oral contraceptives, and ovulation-induction drugs that have traditionally been employed to manage PCOS. Nevertheless, rather than focusing on underlying metabolic dysfunctions, most treatments mainly address symptoms. Micronutrient supplementation has been shown to improve insulin sensitivity, lower oxidative stress, and improve reproductive results when used in conjunction with medication. Additionally, complimentary strategies like dietary pattern optimization, lifestyle change, and new nutraceutical formulations provide synergistic advantages. Combining micronutrients with conventional therapies may enable medication dosage reduction, reduce side effects, and support long-term metabolic health. However, there are currently no standardized clinical guidelines for micronutrient-based therapies, which highlights the necessity of thorough clinical studies and precision-based therapy models (3).

### Limitations of current evidence

Micronutrient-based PCOS treatments are gaining recognition, but there are a few drawbacks to be aware of. Because PCOS is a very diverse syndrome with unique phenotypes, responses to diet therapy can vary. Small sample sizes, brief intervention durations, and varied supplementation protocols with regard to dosage, formulation, and combinations are common limitations of clinical trials. Furthermore, baseline nutritional status is frequently not stratified, which makes it more difficult to interpret results. These considerations emphasize the necessity for long-term, phenotype-specific research and limit the direct translation of findings into standardized treatment advice.

### Future perspective

Despite the growing evidence that nutrition plays a key role in PCOS, current dietary recommendations are broad and not personalized. While emerging evidence supports the concept of precision nutrition in PCOS, its application currently remains largely exploratory and is not yet integrated into routine clinical practice. Future research needs to integrate nutrigenomics, metabolomics and microbiome profiling to develop individualized nutrition plans based on the genetic and metabolic signature of women with PCOS. AI driven modeling can help predict how specific nutrients interact with molecular pathways like PI3K/Akt, NF-κB and Nrf2 and offer precision-based interventions instead of ‘one size fits all’ diets.

Emerging research includes Nutrigenomic mapping, Gut microbiota, Combination therapies, AI/ML in dietary planning, long term RCTs.

## Conclusion

**Figure 9:**
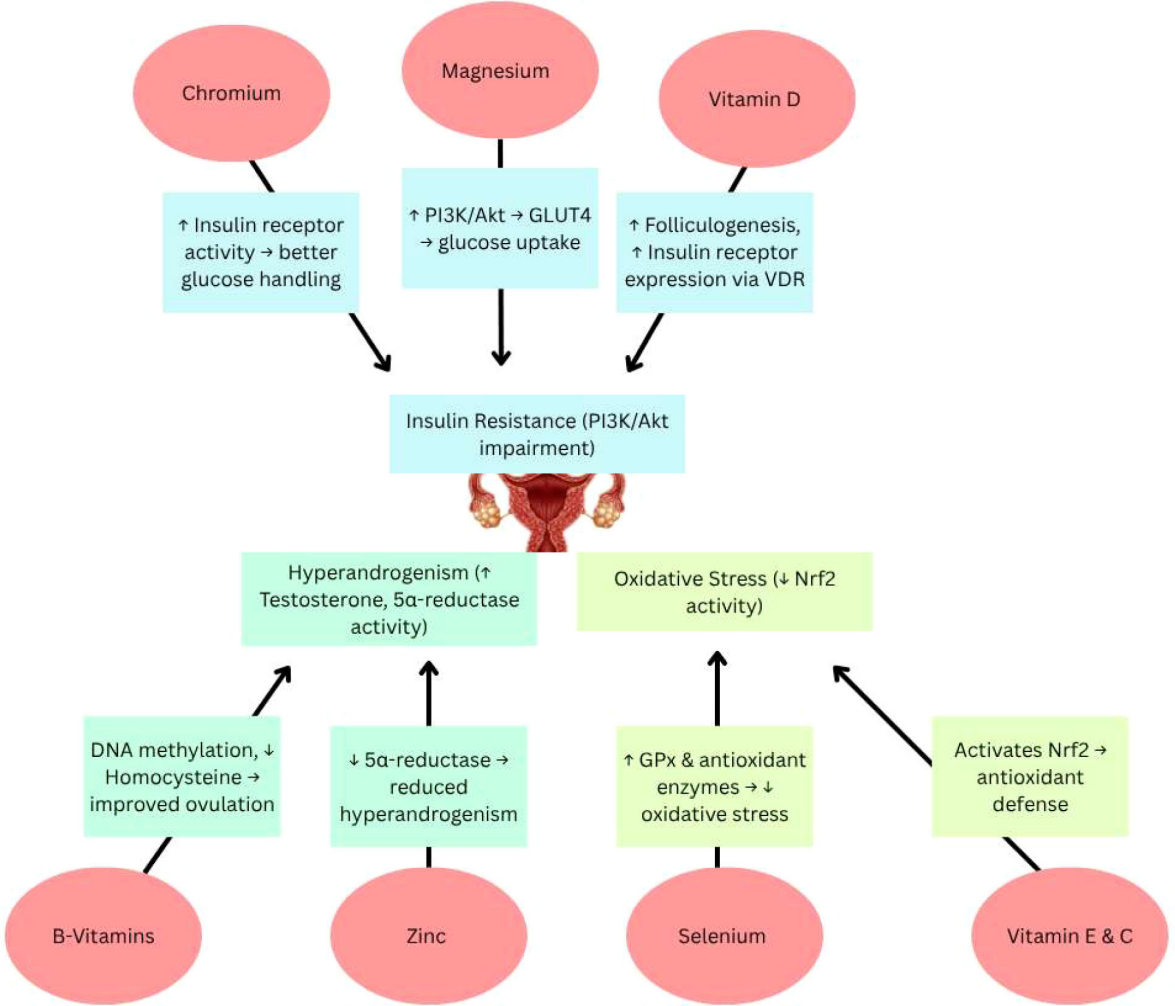
Visual summary of how key micronutrients modulate molecular pathway to improve insulin resistance, hyperandrogenism, inflammation, and oxidative stress in PCOS. PI3K, Phosphoinositide 3-kinase; Akt, Protein kinase B; GLUT4, Glucose transporter type 4; VDR, Vitamin D receptor; GPx, Glutathione peroxidase; Nrf2, Nuclear factor erythroid 2-related factor 2. Symbols: ↑ – Increased; High, ↓ – Decreased/Low.

Micronutrients are emerging as key modulators of the molecular disturbances in PCOS, offering a safe and promising adjunct to conventional therapy. Advancing toward precision nutrition through nutrigenomics, multi-nutrient strategies, and integrative technologies holds the potential to transform PCOS management from symptomatic control to long-term improvement in metabolic and reproductive health (**Figure 9**).
